# Serum Cytokines as Biomarkers in Islet Cell Transplantation for Type 1 Diabetes

**DOI:** 10.1371/journal.pone.0146649

**Published:** 2016-01-11

**Authors:** Cornelis R. van der Torren, Annemarie A. Verrijn Stuart, DaHae Lee, Jenny Meerding, Ursule van de Velde, Daniel Pipeleers, Pieter Gillard, Bart Keymeulen, Wilco de Jager, Bart O. Roep

**Affiliations:** 1 Department of Immunohematology and Blood Transfusion, Leiden University Medical Center, 2333 ZA, Leiden, The Netherlands; 2 Department of Pediatric Immunology, Department of Pediatric Endocrinology and Laboratory of Translational Immunology and Multiplex Core Facility, Wilhelmina Children’s Hospital, University Medical Center Utrecht, 3584 CX, Utrecht, The Netherlands; 3 Dept. of Endocrinology, University Hospital, Katholieke Universiteit Leuven, 3000, Leuven, Belgium; 4 Diabetes Research Center and Academic Hospital, Free University-Vrije Universiteit Brussel (VUB), 1090, Brussels, Belgium; 5 Juvenile Diabetes Research Foundation Center for Beta Cell Therapy in Diabetes; La Jolla Institute for Allergy and Immunology, UNITED STATES

## Abstract

**Background:**

Islet cell transplantation holds a potential cure for type 1 diabetes, but many islet recipients do not reach long-lasting insulin independence. In this exploratory study, we investigated whether serum cytokines, chemokines and adipokines are associated with the clinical outcome of islet transplantation.

**Methods:**

Thirteen islet transplant patients were selected on basis of good graft function (reaching insulin independence) or insufficient engraftment (insulin requiring) from our cohort receiving standardized grafts and immune suppressive therapy. Patients reaching insulin independence were divided in those with continued (>12 months) versus transient (<6 months) insulin independence. A panel of 94 proteins including cytokines and adipokines was measured in sera taken before and at one year after transplantation using a validated multiplex immunoassay platform.

**Results:**

Ninety serum proteins were detectable in concentrations varying markedly among patients at either time point. Thirteen markers changed after transplantation, while another seven markers changed in a clinical subpopulation. All other markers remained unaffected after transplantation under generalized immunosuppression. Patterns of cytokines could distinguish good graft function from insufficient function including IFN-α, LIF, SCF and IL-1RII before and after transplantation, by IL-16, CCL3, BDNF and M-CSF only before and by IL-22, IL-33, KIM-1, S100A12 and sCD14 after transplantation. Three other proteins (Leptin, Cathepsin L and S100A12) associated with loss of temporary graft function before or after transplantation.

**Conclusions:**

Distinct cytokine signatures could be identified in serum that predict or associate with clinical outcome. These serum markers may help guiding patient selection and choice of immunotherapy, or act as novel drug targets in islet transplantation.

## Introduction

Pancreatic islet cell transplantation can cure type 1 diabetes by achieving normoglycaemia with independence of exogenous insulin and near-physiological beta-cell function [1–4]. Beta-cell replacement therapy has been shown to decrease HbA1c levels and the risk of long-term complications, although at the cost of continuous immune suppression [2, 4–8]. Even without complete and long-lasting insulin independence, islet transplantation has a beneficial impact on both the long term outcome in terms of complications as well as on quality of life in patients on the basis of hypoglycaemia unawareness [4, 9]. A key limitation of islet transplantation in most patients is loss of insulin independence over time. Several factors contribute to poor outcome. Firstly, insufficient graft size or quality may impair initial graft function [3], which may lead to beta-cell exhaustion over time. Secondly, inflammatory and immune reactions, such as instant blood mediated immune reaction, recurrent autoimmunity or allograft rejection, can lead to graft destruction [10, 11]. Thirdly, immunosuppressive drugs can affect engraftment and beta-cell function or cause insulin resistance [12]. Knowledge of processes that can affect graft function has expanded over the last years, including insight in markers that may help to predict recurrent autoimmunity [13–15]. Still, major progress may be achieved regarding patient selection and immunosuppression by more detailed understanding and further improved prediction of the processes affecting graft survival [16].

Some insight has been gained in the immunological determinants of islet graft function in humans. The main focus has been on islet specific adaptive immune responses [15, 17–23]. Allograft-specific cytokine profiles *in vitro* have been associated with clinical outcome pointing to IL-10 as a correlate for low alloreactivity and good graft function [21]. Circulating serum proteins, including cytokines and adipokines, have only sparsely been investigated, although one study suggested that levels of certain cytokines (IL-10, IL-13, IL-18, and MIF) were associated with islet graft outcome in patients receiving a kidney and islet transplant [24].

With the emergence of multiplex immunoassay technology, it has become possible to simultaneously investigate a broad spectrum of serum markers in minimal amounts of serum. Our platform has been validated recently to measure a panel of cytokines, adhesion molecules, adipokines, growth factors and various other mediators [25–27].

We designed an explorative study to identify whether the serum secretome (consisting of a panel of cytokines, adipokines, etc.) is affected by transplantation and to investigate whether potential biomarkers that correlate with outcome of islet transplantation can be identified, which may act as targets of intervention therapy or guide patient selection.

## Materials and Methods

### Graft recipients

Thirteen type 1 diabetic patients, transplanted consecutively with standardized islet cell grafts between January 2002 and April 2004, were selected out of a previously described cohort of 21 islet recipients [15]. All recipients were non-uremic and C-peptide negative, had large individual variation of fasted glycaemia (coefficient of variation of pre-breakfast glycaemia >25%), and had one or more signs of diabetic complications (hypoglycaemic unawareness, microalbuminuria or retinopathy). Patient characteristics are depicted in Table 1. Patients were selected on basis of clinical outcome to cover the spectrum from good engraftment (n = 9, reached insulin independence) to insufficient engraftment (n = 4, remained insulin dependent during the first year after transplantation). Three out of nine patients reaching insulin independence had early loss of function (<6 months of insulin independence). The study has been approved by the IRB of the VUB-Free University Brussels and conducted according to the principles expressed in the Declaration of Helsinki. Written informed consent has been obtained of all participants [3].

**Table 1:** Patient and metabolic characteristics. Clinical characteristics of islet cell transplantation patients achieving continued (>6 months), or temporary (<6 months) insulin independence or not achieving (n = 4) insulin independence. Data refer to moment of first islet cell transplantation, unless stated otherwise.

	Reaching Insulin Independence (II)		Insulin Requiring (IR)^a^	p-value
	Continued (C)^a^	Temporary (T)^b^		II-IR / C-T
N	6	3	4	
Gender (M-F)	3–3	3–0	3–1	1.0 / 0.46
Age (yr)	48.5 (41–55)	39, 39, 44	34.5 (31–52)	0.22 / **0.04**
Duration of diabetes	25.5 (14–39)	26, 33, 33	26 (12–33)	0.52 / 0.46
BMI (kg/m2)	26 (17–28)	22, 23, 27	24.5 (23–26)	0.97 / 0.79
HbA1c (%) pre Tx	7.1 (6.5–8.1)	*4*.0*, 6.9, 7.3	7.9 (7.3–8.3)	0.05 / 0.83
CoV pre-breakfast glucose pre Tx	44.0 (36.8–50.0)	35.1, 41.4, 44.1	47.4 (42.2–50.2)	0.14 / 0.33
Number of 2^nd^ transplants	4	1	2	0.93 / 0.46
beta-cell mass (10ˆ6/kg BW)	4.5 (2–9)	4, 4, 6	4 (3–5)	0.16 / 0.54
Time to II (wk)	20 (7–28)	12, 27, 34	N.A.	N.A. / 0.48
Loss of II (wk after Tx)	N.A.	32, 37, 43	N.A.	N.A. / N.A.
HbA1c (%) 1 yr post Tx	6.15 (4.0–6.6)	*4*.1*, 6.9, 5.3	7.1 (5.9–7.8)	0.12 / 0.86
CoV pre-breakfast glucose 1 yr post Tx	9.9 (7.7–13.6)	9.7, 14.1, 19.9	29.9 (17.2–31.2)	0.0002 / 0.12

Tx: islet transplantation; CoV: coefficient of variation; N.A. not applicable; BW: body weight. P-values represent Students t-test for numerical and Fisher’s exact test for binominal data.

^a^median (range).

^b^individual values.

*Unreliable measurement, excluded from statistical analysis.

### Transplant procedure and follow-up

Islet cell grafts were injected into the portal vein under anti-thymocyte globulin (ATG, Fresenius Hemocare, WA, USA) induction therapy and maintenance immune suppression with myco-phenolate mofetil and tacrolimus [3]. Maintenance immune suppression was continued throughout the first year irrespective of graft function. Blood samples were taken before transplantation and at one year (median 53 weeks; range 47–60) after transplantation in serum tubes containing silicate granulate. Serum was aliquotted and stored at -80°C until analysis.

### Serum mediators

Measurement of 94 serum markers was performed using in house developed and validated multiplex immunoassay (laboratory of translational immunology, University Medical Center Utrecht) based on xMAP technology (Luminex Austin TX USA) [25, 27]. An overview of the markers is shown in Table 2 (further detail in S1 Table). For the measurement of biomarkers naturally occurring in high concentrations, samples were diluted either 1:100 (Adipsin, Cathepsin S, Cathepsin L, Chemerin, Leptin, PAI-1, CCL18, RBP-4, Resistin, SAA-1, TIMP-1, TPO, sCD14, sICAM and sVCAM) or 1:1000 (Adiponectin). Acquistion was performed with a Biorad FlexMap3D system using Xponent 4.2 software. Serum values were calculated using Bio-Plex Manager 6.1.1.

**Table 2:** Overview of serum markers. Ninety-four serum markers were measured consisting of cytokines, chemokines, adipokines, growth factors and other immune related proteins. Cytokines with >20% missing values are indicated with (^§^).

Cytokines		Chemokines	Adipokines	Growth factors	Other
IL-1α	IL-18	CCL1^§^	Adiponectin	BDNF	FAS
IL-1β	IL-21	CCL2	Adipsin	EGF	FAS-L
IL-1Ra^§^	IL-22^§^	CCL3	Cathepsin B	G-CSF	Granzyme B
IL-2^§^	IL-23	CCL4	Cathepsin L	GM-CSF	IL-1RII
IL-3^§^	IL-25	CCL7	Cathepsin S	HGF	IL-18BPA
IL-4	IL-27	CCL11	Chemerin	M-CSF	KIM_1
IL-5^§^	IL-33	CCL17	Leptin	NGF	MMP-8
IL-6^§^	IFNα^§^	CCL18	Omentin	SCF	OPG
IL-7^§^	IFNβ	CCL19	PAI-1	sICAM	OPN
IL-9	IFNγ^§^	CCL22	RBP-4	sVCAM	S100A12
IL-10	LIF	CCL27^§^	Resistin	VEGF	sCD14
IL-11	MIF	CXCL5	SAA-1		sCD25
IL-12	OSM	CXCL8	TIMP-1		sCD163
IL-13	TNFα^§^	CXCL9	Trombopoietin		sIL-6R
IL-15	TNFβ^§^	CXCL10			sPD-1
IL-16	TSLP	CXCL13			sSCF-R
IL-17^§^		XCL-1			TNF-RI
					TNF-RII
					TREM-1

### Statistical analyses

Descriptive statistics are reported as median with range. For analysis, data was normalized by log transformation and out-of-range values above detection limit were set to maximum of standard curve; values below the minimum of the standard curve that could not be reliably extrapolated were set to the lowest detected value. Student’s t-test (paired if applicable) with Welch correction for variance was used for numerical values to ensure quantitative effect analysis, realizing that the data set is too small to ensure normal distribution. Fisher’s exact test was used for binominal data. The sized-limited dataset did allow further selection by multiple testing correction.

For analysis of changes over time, samples continuously out-of-range were excluded. All statistics and figure plotting were performed with R v3.0.0, aided by packages Stats, Plotrix, Graphics, VennDiagram and Heatmap.plus (R Core Team (2013). R: A language and environment for statistical computing. R Foundation for Statistical Computing, Vienna, Austria. URL: http://www.R-project.org/). P values of <0.05 were considered significant.

## Results

### Metabolic outcome

Patients were categorized by graft function during the first year after transplantation; nine out of 13 patients reached insulin independence (good engraftment) while four patients remained insulin requiring (insufficient engraftment). These groups did not differ regarding clinical and graft characteristics except for HbA1c, which tended to be lower before transplantation in patients reaching insulin independence (p = 0.05). HbA1c improved in all patients with good engraftment and in two of four patients with insufficient engraftment. Pre-breakfast glucose variability improved in all patients, but much more pronounced in patients who reached insulin independence. Pre-breakfast glucose variability was significantly different in insulin independent from insulin requiring patients one year after transplantation (p = 0.0002). Of nine insulin independent patients, three had temporary insulin independence during the first year after transplantation while six continued to be insulin independent (Table 1).

### Detection of Serum markers

A total of 94 serum markers were measured (Table 2), of which four (OSM, CCL1, CCL27 and NGF) were undetectable in all patient samples and one (Omentin) did not show any variability. Therefore, these were excluded from further analysis. Furthermore, 16 other markers showed more than 20% of values out of measurement range; these values were substituted as described in the methods section. Out-of-range values were evenly distributed between both groups, except for leukaemia inhibitory factor (LIF), which was mainly detected in patients with good engraftment.

### Impact of transplantation and immunosuppression on serum marker levels

**Fig 1:**
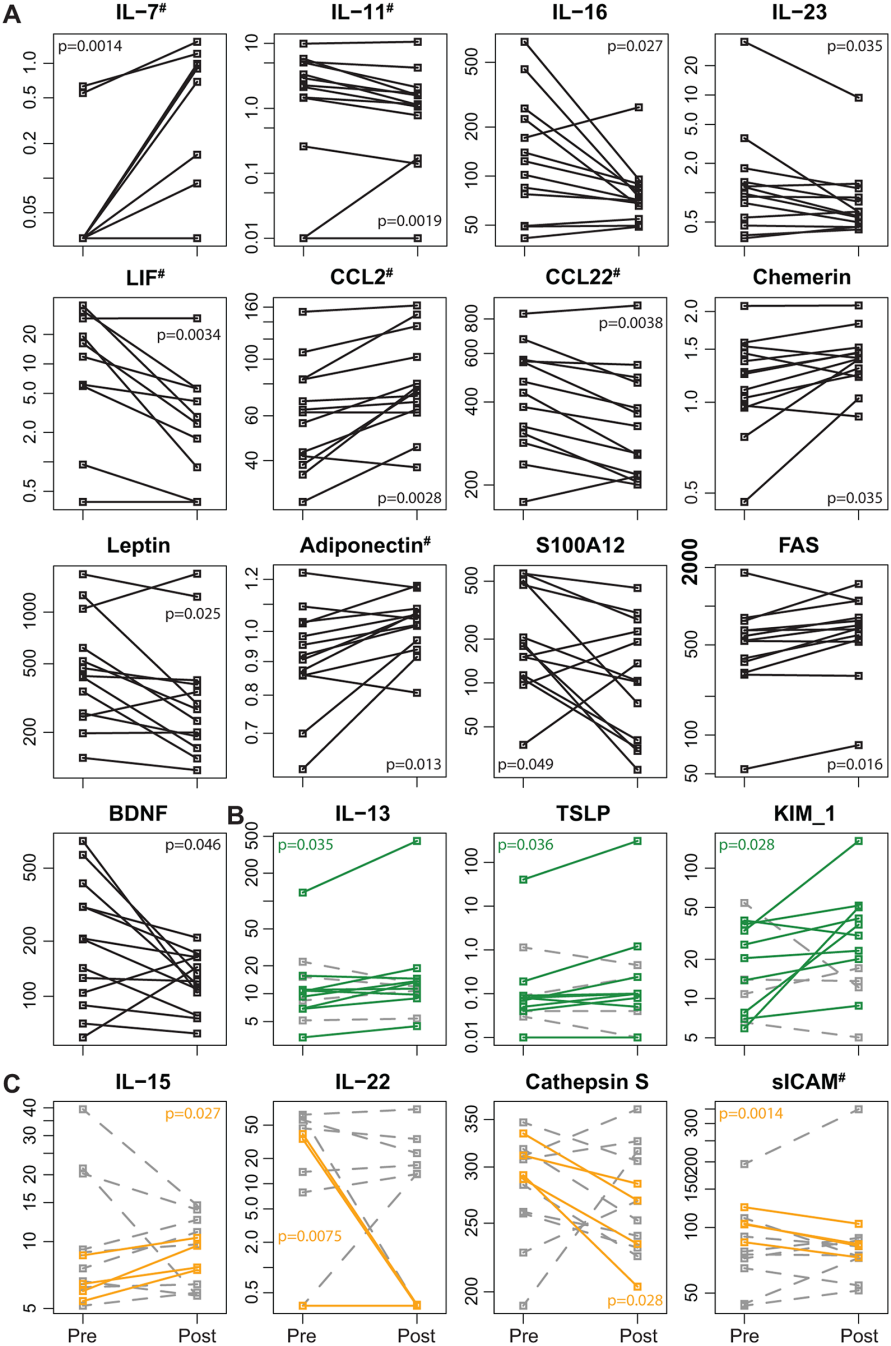
Serum markers changing by transplantation and immunosuppression. Serum marker levels that significantly change from pre islet cell transplantation to one year post transplantation are depicted (Student’s t-test, p<0.05): for all patients (A), for patients with good engraftment (B, green) and for patients with insufficient engraftment (C, orange). Serum levels are in pg/ml for: IL-7, IL-11, IL-13, IL-15, IL-16, IL-22, LIF, TSLP, CCL2, FAS, BDNF, KIM-1; ng/ml for: IL-23, CCL22, Cathepsin S, sICAM, S100A12; in ug/ml for: Chemerin, Leptin; mg/ml for: Adiponectin. Samples out-of-range at both time points were excluded from statistical analysis.

Serum marker levels pre- and post-transplantation were studied irrespective of graft function. Notably, serum protein levels varied widely among individuals while levels of individual patients, comparing pre- and post-transplantation samples, remained markedly stable in spite of transplantation and immunosuppression (inter-patient variability was larger than intra-patient variability for all analytes). Nevertheless, 13 markers changed with intervention: IL-7, CCL2, Adiponectin, Chemerin and FAS increased, while IL-11, IL-16, IL-23, LIF, CCL22, Leptin, BDNF and S100A12 decreased during the first year after transplantation (Fig 1A).

Taking graft function into account while analyzing the effect of intervention, another three markers (IL-13, TSLP and KIM-1) were identified in patients with good engraftment and another four markers (IL-15, IL-22, cathepsin S and sICAM) in patients with insufficient engraftment. IL-13, TSLP and KIM-1 increased after transplantation in patients reaching insulin independence (Fig 1B); while IL-15 increased and IL-22, Cathepsin S and sICAM decreased in insulin requiring patients (Fig 1C).

### Identifying biomarkers of islet transplantation outcome

**Fig 2:**
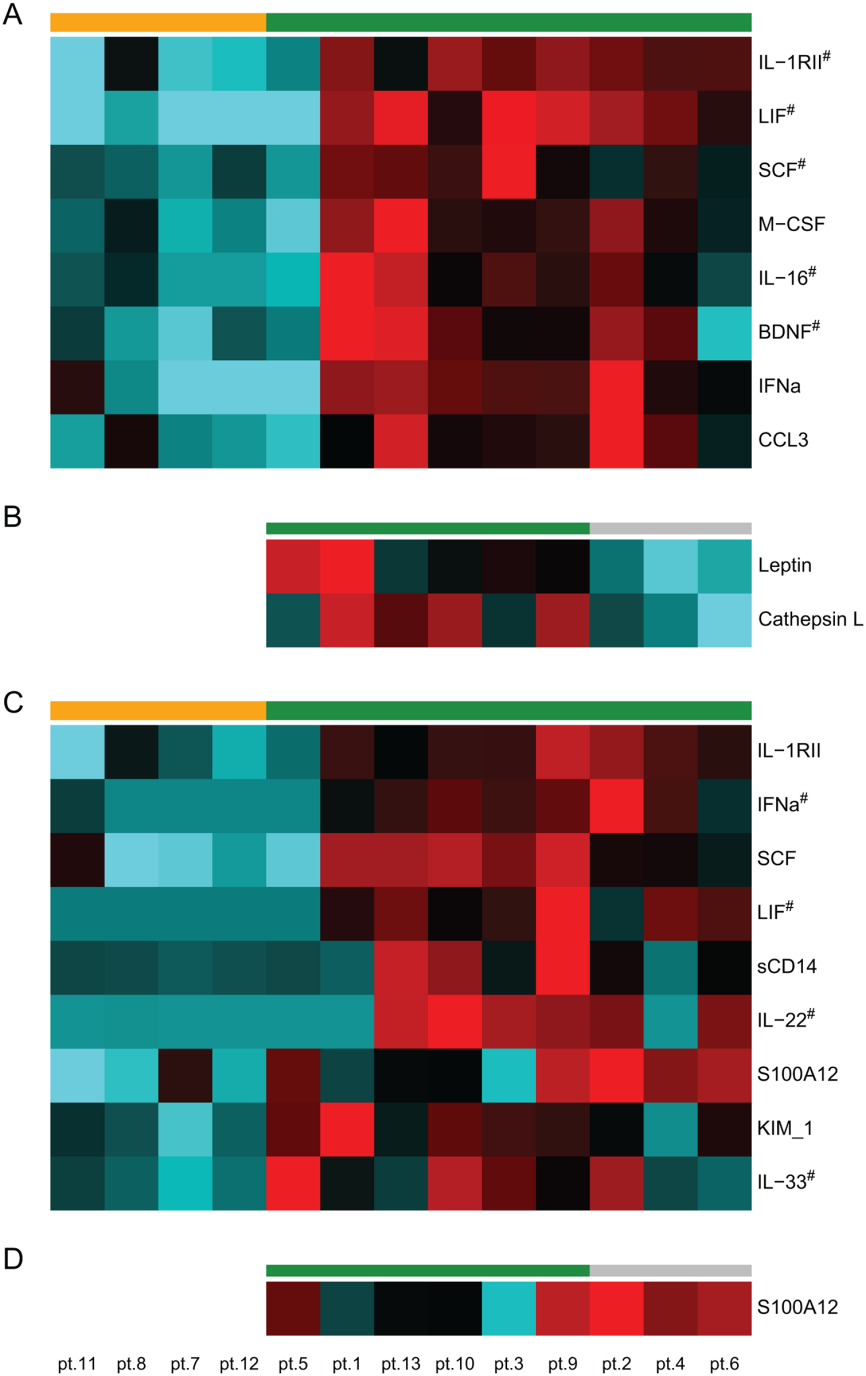
Serum marker levels before or after islet transplantation that could differentiate between good and poor graft function. Heatmap representation of significantly different serum markers between patients with good graft function (green) and patients with poor graft function (orange) before (A&B) or 1 year after (C&D) transplantation (Student’s t-test, p<0.05). In the group of patients with good graft function, those with continued insulin independence (green) and temporary insulin independence (grey) were subdivided (B & D). Heatmap gradient represents min to max (cyan—black—red) normalized serum titers.

Firstly, serum marker levels before transplantation were compared between individuals with good and insufficient engraftment. Eight markers (IL-16, IFN-α, LIF, CCL3, BDNF, M-CSF, SCF and sIL-1RII) were higher in individuals with good engraftment, while no markers were lower (Fig 2A). In addition, we investigated whether serum marker levels before transplantation in patients with good engraftment differed between patients with and without gradual loss of graft function during the first year. Leptin and Cathepsin L were higher in individuals with continued insulin independence (Fig 2B).

Next, we analyzed whether serum levels one year after transplantation reflect graft function. Patients with good engraftment had higher levels of IL-22, IL-33, IFN-α, LIF, SCF, IL-1RII, KIM-1, S100A12 and sCD14, than patients with insufficient engraftment (Fig 2C). Patients with continued insulin independence had lower S100A12 than patients with transient insulin independence after initial good graft function (Fig 2D).

**Fig 3:**
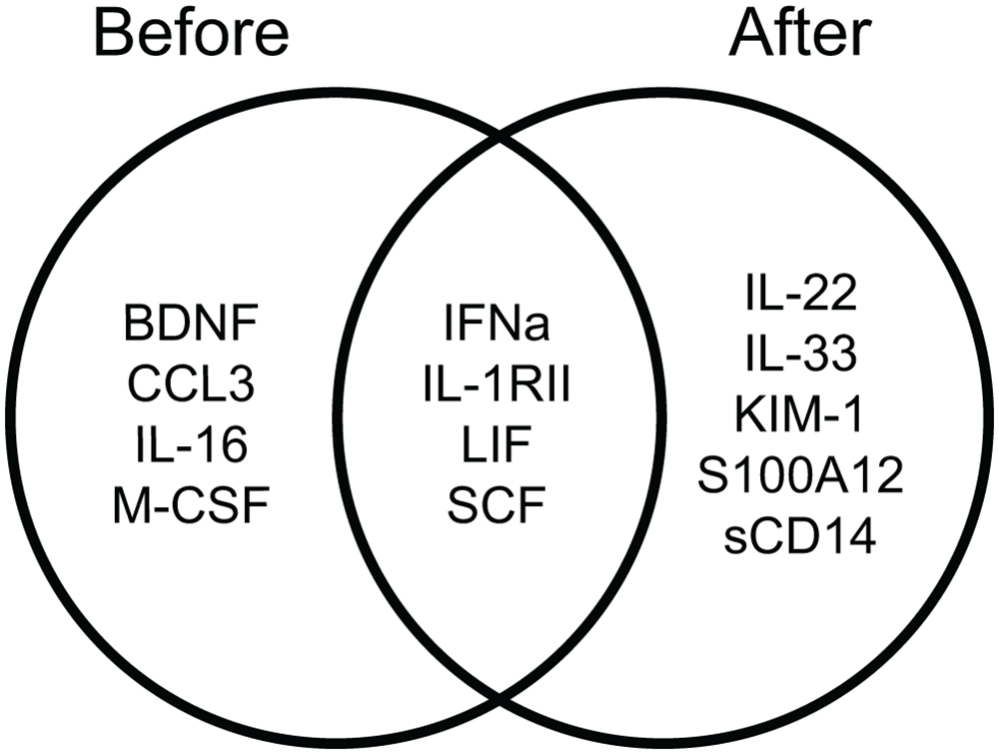
Overlap of serum markers correlating with outcome of islet transplantation. Venn diagram showing serum markers that predict or correlate with clinical outcome (good or poor graft function) 1 year after islet transplantation.

In total 13 serum markers were identified that associated with reaching insulin independence after islet transplantation, of which four (IFN-α, LIF, SCF, IL-1RII) associated with insulin independence before as well as one year after transplantation (Fig 3).

## Discussion

Upon intraportal islet infusion, islets come in direct contact with serum proteins. Although many of these proteins are related to immune responses and several have been described to affect beta-cell survival directly, these have been poorly studied in islet transplantation [28, 29]. Therefore, we investigated whether serum markers relate to clinical outcome and describe the effect of islet transplantation on these markers.

Thirteen patients were selected from a cohort of islet recipients receiving a standardized graft under a stringent protocol [3, 15]. Before and after transplantation we tested a large serum marker panel in a multiplex immunoassay. We reasoned this unbiased approach would allow us to study changes in immune and endocrine homeostasis after islet transplantation and immunosuppression, thereby identifying potential biomarkers of islet graft survival that may help understand and improve transplantation protocols or patient selection for individualized care.

Choosing a homogeneous set of patients transplanted in a short time frame decreased sample size from this unavoidably limited islet transplant cohort, but minimized variation in transplantation procedures, sample handling, and the chance that technical alterations affect outcome [26]. This limited data set did not allow multiple testing correction to verify individual cytokines as biomarkers of outcome. Nonetheless, groups of serum markers were identified that associated with transplantation and transplantation outcome.

The first year after allograft transplantation most serum markers were stable within patients, while varying more widely between patients. This was despite the use of immunosuppression in all patients and major improvement of blood glucose homeostasis in most patients. This observation does not necessarily imply that the current immune suppressive protocol has no effect on investigated analytes with potential clinical or therapeutic value, as local effects in the lesions may not be reflected in serum or may only be temporary. Observations on CCL2 may underline differences between local and systemic effects. CCL2 release by human islets prepared for transplantation was shown to positively associate with release of other pro-inflammatory mediators (IL-6, CXCL-8) by islets, with inflammatory markers in serum immediately after transplantation and to correlate inversely with transplantation outcome [29, 30]. Furthermore, high islet CCL2 release was associated with higher parameters of inflammation in recipients. We observed that *systemic* pre-transplantation CCL2 levels did not predict graft function; however our finding that CCL2 levels increase during the first year after transplantation corroborates previous observations on the role of CCL2.

The role of glucose on serum marker stability is of interest. Increased serum glucose levels are known to affect several inflammatory proteins in the circulation [31, 32]. On the other hand, increased pro-inflammatory cytokine levels such as IL-1β, TNF-α and IFN-γ have been shown *in vitro* to decrease glucose oxidation, activity of glycolytic enzymes and inhibit insulin secretion, thus creating a vicious loop [33]. In our cohort, HbA1c levels tended to be lower before transplantation in patients reaching insulin independence, reflecting lower glucose levels and thereby possibly affecting serum marker levels. However, a major influence of blood glucose on measured markers seems unlikely, since few serum proteins changed significantly while glucose control improved in all patients and HbA1c levels did not differ with clinical outcome one year after transplantation.

The second aim of this study was to identify possible biomarkers of islet graft survival. We identified 13 serum markers that correlated with insulin independence in the first year after islet transplantation, of which eight associated with outcome before transplantation. Serum markers that may predict outcome represented various categories of inflammatory mediators; mostly growth and differentiation factors (LIF, BDNF, M-CSF and SCF), which may promote graft survival, and mediators of innate immunity (IL-16, IFN-α, CCL3, and IL-1RII), which may stimulate regulatory responses or prevent recruitment of harmful cells by masking the local cytokine gradient.

Of the factors identified by this study that correlate with outcome, LIF associated most clearly, but has not been extensively investigated. Yet, LIF has previously been identified to regulate beta-cell mass and promote islet cell survival and allograft tolerance via Treg induction [34–37]. Furthermore, targeted LIF delivery in combination with nano-PEG-encapsulation of the islet graft was shown to give superior graft survival *in vivo* [38]. Our finding that LIF was predominantly detectable in patients with good engraftment is in concordance with previous results and underlines the role of LIF as possible mediator influencing beta-cell mass.

At one year after transplantation, several growth and differentiation factors retained their discriminative capacity (LIF, IFN-a, SCF, IL-1RII) (Fig 3). Moreover, IL-22 (member of the IL-10 family); IL-33 (member of the IL-1 family) and molecules of the innate immune system (S100A12, sCD14 and kidney injury molecule 1 (KIM-1)) distinguished patients with good and poor graft function. For example, KIM-1 is a molecule of which urinary levels are associated with tubular kidney damage [39]. Urinary KIM-1 levels are elevated in type 1 diabetic patients irrespective of albuminuria, suggesting tubular damage may be present at an early stage [40]. In kidney transplantation, serum KIM-1 levels were powerful predictors of acute rejection [41]. In contrast, we found serum KIM-1 levels one year after transplantation to be higher in patients with good outcome, which may indicate tubular damage that is not reflected in serum creatinine rise and could be caused by greater sensitivity to (side)effects of immunosuppressive drugs. Secondly, IL-33 is able to activate cells of both the innate and adaptive immune system. In addition, depending on the disease, IL-33 can either promote the resolution of inflammation or drive disease pathology [42]. Furthermore, cytotoxic T cells express high levels of the IL-33 receptor ST2 and elevated levels of decoy receptor soluble ST2 associate with Type 2 Diabetes [43, 44]. In this study, higher IL-33 levels were associated with retained graft function after islet transplantation. Thirdly, IL-22 is thought to play a role in coordinating adaptive and innate immune responses. IL-22 has both pro-inflammatory and tissue-protective properties depending on the context in which it is expressed. Th17 cells, specifically implicated in autoimmune diseases including type 1 diabetes, can secrete IL-22 and IL-17A, which has been identified as a factor promoting the pro-inflammatory effect of IL-22 [45–48]. In our study, IL-22 levels post-transplant were higher in patients with good engraftment, while IL-17 levels were very low in all but one patient. This suggests a tissue-protective role for IL-22, possibly, influenced by the immunosuppressive regimen.

Several of the markers measured in this study have been described previously as predictors of islet cell transplantation outcome after *in vitro* culture of islets (CCL2 and CXCL8) or after stimulation of immune cells (IL-10) [21, 30, 49]. None of these markers measured in serum correlated with outcome of islet cell transplantation in this study. Possibly, local production of these factors is not sufficient to considerably alter circulating levels. Furthermore, increased levels of CXCL8 have been reported immediately after islet infusion. It is therefore conceivable that any change in production of these factors is temporary and has returned to normal one year after transplantation.

Previously, IL-13 and IL-18 were described to be prospectively associated with subsequent loss of graft function in islet *after* kidney transplantation after correction for suggested confounders [24]. In addition, elevated MIF concentrations were associated with subsequent graft loss in *simultaneous* islet and kidney transplantation, while IL-10 was lower in patients losing graft function [24]. In our study, in the context of islet transplantation without kidney transplantation, we could not confirm a predictive role for IL-18 or MIF, nor did we confirm IL-10 to correlate with engraftment. In addition, IL-13 did not differentiate between good and poor engraftment overall, although an increase in IL-13 during the first year correlated with retained graft function within the group of patients with good engraftment.

In conclusion, we identified several serum markers that may give insight in the fate of islet allografts. In addition, these markers can be further investigated for their potential as biomarkers to guide individualized therapy. Our observations warrant further studies to validate these markers and their possible role in predicting long-term graft survival.

## Supporting Information

S1 Fig Heatmap representation of all data. Colors are scaled per analyte on the complete data set from low (cyan) to mean (black) to high (red). Patients are grouped: insulin requiring (orange), insulin independent (green) and temporary insulin independent (grey). Analytes were not reordered.
(PDF)

S1 Study Data Study data overview. (XLS)

S1 Table Table with results of all cytokines. Serum marker levels in islet cell transplantation patients pre- and 1 year post-transplantation are depicted per group. Values are depicted as median (range) in pg/ml, except when indicated: ng/ml^A^, μg/ml^B^ or mg/ml^C^. Out-of-range levels are depicted as zero or maximum of measurement range, indicated with (#). Cytokines with >20% missing values are indicated with (^§^).
(DOC)
