# Supplementary material for: Serum Cytokines as Biomarkers in Islet Cell Transplantation for Type 1 Diabetes
Source: PLoS One. 2016 Jan 11;11(1):e0146649. doi: 10.1371/journal.pone.0146649 (PMC4713434; doi:10.1371/journal.pone.0146649)
Supplement: S1 Fig — Colors are scaled per analyte on the complete data set from low (cyan) to mean (black) to high (red). Patients are grouped: insulin requiring (orange), insulin independent (green) and temporary insulin independent (grey). Analytes were not reordered. (PDF) [file pone.0146649.s001.pdf]

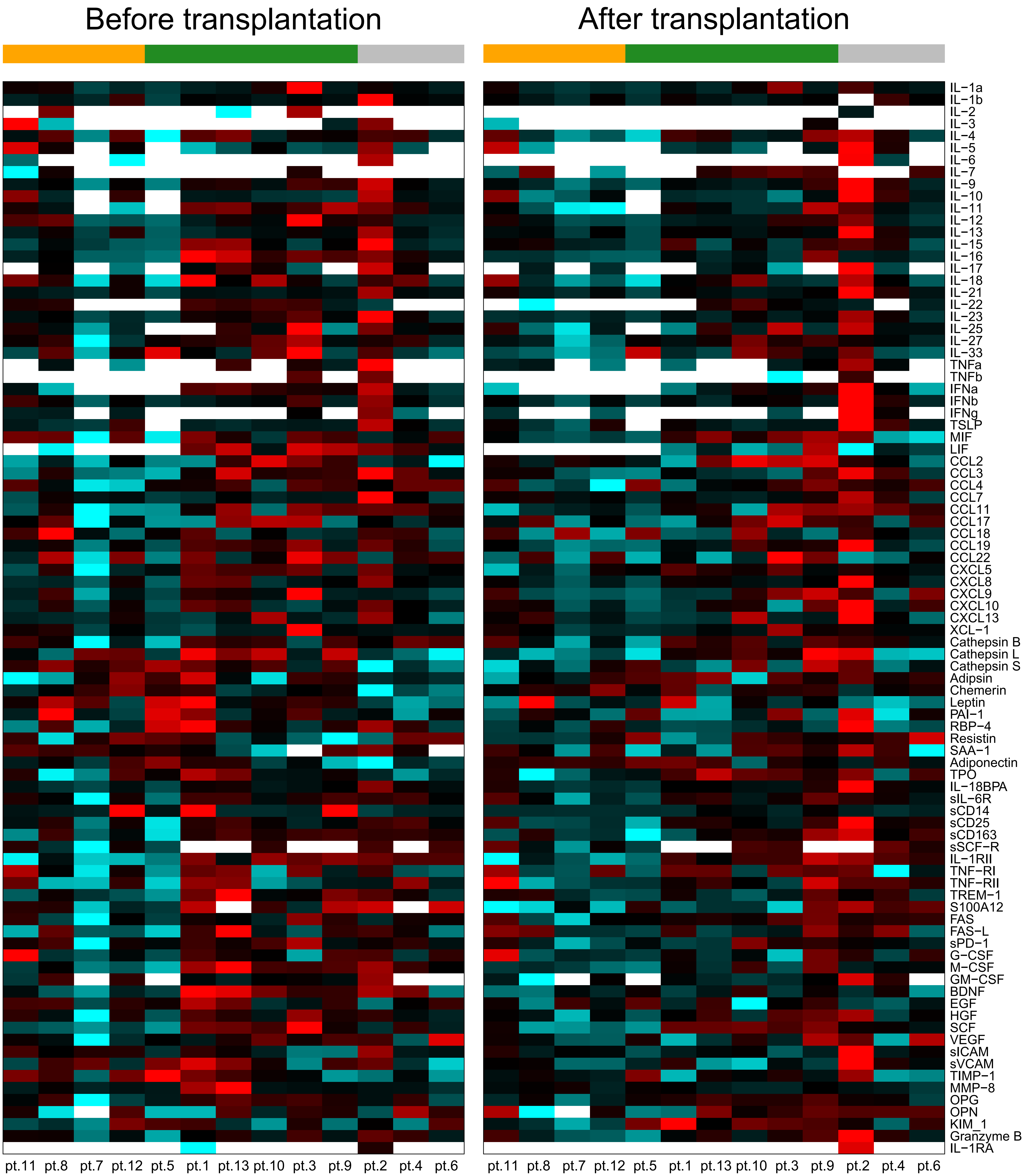

Heatmap representation of all data. Colors are scaled per analyte on the complete data set from low (cyan) to mean(black) to high (red). Patients are grouped: insulin requiring (orange), insulin independent (green) and temporary insulin independent (grey). Analytes were not reoredered.
