# Supplementary material for: Serum Cytokines as Biomarkers in Islet Cell Transplantation for Type 1 Diabetes
Source: PLoS One. 2016 Jan 11;11(1):e0146649. doi: 10.1371/journal.pone.0146649 (PMC4713434; doi:10.1371/journal.pone.0146649)
Supplement: S1 Table — Serum marker levels in islet cell transplantation patients pre- and 1 year post-transplantation are depicted per group. Values are depicted as median (range) in pg/ml, except when indicated: ng/mlA, μg/mlB or mg/mlC. Out-of-range levels are depicted as zero or maximum of measurement range, indicated with (#). Cytokines with >20% missing values are indicated with (§). (DOC) [file pone.0146649.s003.doc]

## Supporting table: Overview of serum marker levels

Serum marker levels in islet cell transplantation patients pre- and 1 year post-transplantation are depicted per group. Values are depicted as median (range) in pg/ml, except when indicated: ng/ml^A^, μg/ml^B^ or mg/ml^C^. Out-of-range levels are depicted as zero or maximum of measurement range, indicated with (#). Cytokines with >20% missing values are indicated with (^§^).

|  | Reaching Insulin Independence | | | | Insulin Requiring | | |
| --- | --- | --- | --- | --- | --- | --- | --- |
|  | Continued Insulin Independence | | Temporary Insulin Independence | | |  | |
| **Cytokines** | Pre | Post | Pre | Post | | Pre | Post |
| IL-1α | 2.51 (1.83 - 27.9) | 2.59 (1.89 - 10.1) | 2.9 (2.39 - 3.57) | 2.77 (2.51 - 5.77) | | 2.84 (1.69 - 3.75) | 2.17 (1.91 - 3.7) |
| IL-1β | 1.81 (0.95 - 1.99) | 1.76 (1.14 - 2.36) | 1.98 (1.84 - 42) | 4 (2.02 - 2070#) | | 1.52 (1.31 - 4.01) | 1.86 (1.21 - 2.19) |
| IL-1Ra^§^ | 0 (0 - 19.9) | 0 (0 - 0) | 0 (0 - 148) | 0 (0 - 540) | | 0 (0 - 0) | 0 (0 - 0) |
| IL-2^§^ | 0 (0 - 7.28) | 0 (0 - 0) | 0 (0 - 0) | 0 (0 - 2.11) | | 0 (0 - 5.46) | 0 (0 - 0) |
| IL-3^§^ | 0 (0 - 8.77) | 0 (0 - 12.4) | 0 (0 - 28.8) | 0 (0 - 0) | | 1.54 (0 - 69.6) | 0 (0 - 3.07) |
| IL-4 | 0.94 (0.51 - 1.2) | 0.935 (0.57 - 1.24) | 1.05 (0.82 - 1.07) | 0.98 (0.78 - 1.38) | | 0.995 (0.67 - 1.11) | 0.785 (0.65 - 1.14) |
| IL-5^§^ | 1.38 (0 - 3.38) | 1.48 (0 - 2.64) | 1.38 (0 - 14.6) | 4.43 (0 - 47.1) | | 3.77 (0 - 32.6) | 0.32 (0 - 24.1) |
| IL-6^§^ | 0 (0 - 0) | 0 (0 - 0) | 0 (0 - 1160) | 79.7 (0 - 2680) | | 4.86 (0 - 51.8) | 0 (0 - 0) |
| IL-7^§^ | 0 (0 - 0.63) | 0.825 (0 - 1.21) | 0 (0 - 0) | 0 (0 - 0.9) | | 0.015 (0 - 0.55) | 0.125 (0 - 1.54) |
| IL-9 | 41.4 (3.05 - 77.4) | 19.4 (4.68 - 62.7) | 25.5 (15 - 696) | 52.5 (13.3 - 1600) | | 20.4 (2.49 - 26.5) | 12.2 (3.33 - 21.8) |
| IL-10 | 20.9 (0 - 37.6) | 20.9 (0 - 45.6) | 43.4 (34.7 - 2090) | 181 (35.3 - 8720) | | 88.7 (0 - 836) | 28.4 (4.06 - 744) |
| IL-11 | 4.2 (0 - 9.9) | 1.36 (0.01 - 10.6) | 2.91 (1.47 - 5.2) | 1.58 (1.16 - 4.22) | | 0.855 (0 - 2.26) | 0.475 (0.14 - 1.7) |
| IL-12 | 11.8 (5.7 - 56.4) | 9.84 (6.77 - 15.3) | 9.06 (8.88 - 15.6) | 9.67 (7.83 - 25.8) | | 12.4 (6.08 - 21.4) | 8.93 (6.72 - 13.3) |
| IL-13 | 10 (3.37 - 15.6) | 12.4 (4.46 - 14.5) | 10.6 (6.87 - 123) | 18.8 (8.9 - 445) | | 11.8 (5.14 - 22.1) | 11.4 (5.39 - 12.8) |
| IL-15 | 9.09 (5.15 - 21.4) | 8.07 (5.71 - 13.9) | 7.58 (6.64 - 39.4) | 11 (5.71 - 14.6) | | 6.22 (5.4 - 8.68) | 8.62 (7.46 - 10.4) |
| IL-16 | 198 (41.6 - 668) | 82 (48.9 - 265) | 124 (85 - 260) | 79.3 (68.2 - 84.7) | | 63.4 (49 - 102) | 62.6 (49.6 - 71.2) |
| IL-17^§^ | 0.13 (0 - 0.68) | 0.01 (0 - 0.2) | 0.25 (0 - 4.01) | 0.08 (0 - 8.67) | | 0.02 (0 - 0.23) | 0.06 (0 - 0.15) |
| IL-18 | 17.9 (4.54 - 80.3) | 14.7 (4.39 - 36.2) | 23.8 (11.2 - 31.8) | 16.7 (9.63 - 52.1) | | 20 (4.44 - 34.7) | 11.5 (6.33 - 41.1) |
| IL-21^A^ | 1.73 (0.616 - 1.98) | 1.78 (0.667 - 2.62) | 1.98 (1.7 - 45.7) | 4.73 (1.7 - 246) | | 1.62 (1 - 3.26) | 1.73 (1.08 - 3.61) |
| IL-22^§^ | 51.8 (0 - 66.6) | 19.9 (0 - 77.4) | 0 (0 - 7.87) | 13 (0 - 13.2) | | 17.1 (0 - 39.3) | 0 (0 - 0.36) |
| IL-23^A^ | 1.21 (0.367 - 3.61) | 0.724 (0.421 - 1.11) | 1.16 (0.461 - 35.1) | 1.24 (0.445 - 9.37) | | 0.67 (0.342 - 0.976) | 0.528 (0.45 - 0.64) |
| IL-25 | 138 (0 - 10300) | 160 (0 - 3990) | 319 (251 - 1470) | 236 (231 - 4310) | | 199 (28.5 - 457) | 128 (11.3 - 569) |
| IL-27 | 505 (291 - 2110) | 425 (279 - 1140) | 438 (208 - 571) | 306 (219 - 398) | | 277 (20.6 - 549) | 172 (38.2 - 277) |
| IL-33 | 7.7 (3.41 - 27.1) | 6.49 (3.41 - 18.5) | 3.76 (2.51 - 8.63) | 3.27 (2.76 - 10.9) | | 4.7 (1.73 - 9.39) | 2.71 (1.76 - 3.37) |
| IFNα^§^ | 12.7 (0 - 20.4) | 8.3 (0 - 13.3) | 7.68 (5.68 - 45.5) | 9.56 (2.28 - 79.4) | | 1.03 (0 - 8.27) | 0 (0 - 1.94) |
| IFNβ | 183 (118 - 673) | 192 (105 - 286) | 238 (124 - 1730) | 404 (147 - 7950) | | 267 (66 - 571) | 184 (71.5 - 244) |
| IFNγ^§^ | 0 (0 - 306) | 0 (0 - 104) | 64 (0 - 1790) | 336 (0 - 10000) | | 195 (0 - 202) | 43.2 (0 - 150) |
| LIF | 22.7 (0 - 39.5) | 2.66 (0 - 29.2) | 11.8 (6.14 - 19.1) | 4.15 (0.88 - 5.6) | | 0 (0 - 0.94) | 0 (0 - 0) |
| MIF^A^ | 5.83 (0.825 - 10.2) | 7.74 (2.18 - 13.4) | 4.01 (3.22 - 7.11) | 1.36 (0.963 - 9.58) | | 8.35 (0.725 - 9.44) | 2.74 (2.38 - 4.2) |
| OSM | 0 (0 - 0) | 0 (0 - 0) | 0 (0 - 0) | 0 (0 - 0) | | 0 (0 - 0) | 0 (0 - 0) |
| TNFα^§^ | 5.67 (0 - 27.6) | 2.68 (0 - 15.6) | 0 (0 - 202) | 14.2 (0 - 80.5) | | 1.8 (0 - 13) | 7.74 (0 - 12.3) |
| TNFβ^§^ | 0 (0 - 76) | 0 (0 - 24.9) | 0 (0 - 82.9) | 0 (0 - 68.9) | | 0 (0 - 0) | 0 (0 - 0) |
| TSLP | 0.075 (0 - 0.09) | 0.1 (0 - 0.24) | 0.19 (0.04 - 40.3) | 1.2 (0.08 - 307) | | 0.065 (0.03 - 1.14) | 0.14 (0.01 - 0.45) |
| **Chemokines** |  |  |  |  | |  |  |
| CCL1^§^ | 0 (0 - 0) | 0 (0 - 0) | 0 (0 - 0) | 0 (0 - 0) | | 0 (0 - 0) | 0 (0 - 0) |
| CCL2 | 83.3 (41.7 -153) | 118 (37.6 - 162) | 56.1 (27.5 - 63.4) | 68 (45.2 - 80.1) | | 50.2 (35.2 - 68.4) | 73.5 (61.7 - 78) |
| CCL3 | 121 (94.2 - 147) | 109 (99 - 128) | 130 (113 - 154) | 125 (108 - 152) | | 101 (99 - 120) | 111 (100 - 118) |
| CCL4 | 209 (107 - 258) | 200 (95.6 - 323) | 282 (171 - 282) | 220 (192 - 244) | | 113 (54.3 - 260) | 120 (48.1 - 251) |
| CCL7 | 11.7 (8.18 - 16.1) | 13.2 (9.5 - 21.8) | 16.3 (7.96 - 324) | 27.2 (7.5 - 139) | | 13.8 (6.57 - 19.1) | 17.5 (14 - 22.2) |
| CCL11 | 52.2 (13.7 - 97) | 42.2 (11.8 - 112) | 65.8 (43.8 - 69.5) | 71.6 (50.9 - 102) | | 16.3 (6.46 - 44.3) | 27.9 (10.2 - 32.6) |
| CCL17 | 192 (59.6 - 395) | 182 (58 - 459) | 138 (107 - 152) | 191 (81.3 - 213) | | 136 (32.6 - 238) | 133 (60.3 - 235) |
| CCL18^A^ | 590 (82.6 - 7570) | 227 (45 - 29500) | 3150 (84.4 - 3900) | 848 (270 - 3900) | | 2250 (53.8 - 457000) | 1960 (12.4 - 38300) |
| CCL19 | 5.79 (1.51 - 14.2) | 3.62 (1.14 - 7.49) | 5 (1.57 - 9.87) | 2.59 (1.59 - 44.2) | | 3.34 (0.95 - 4.35) | 1.52 (0.83 - 3.48) |
| CCL22 | 471 (238 - 836) | 345 (201 - 896) | 432 (285 - 474) | 257 (218 - 380) | | 448 (174 - 676) | 365 (216 - 491) |
| CCL27^§^ | 0 (0 - 0) | 0 (0 - 0) | 0 (0 - 0) | 0 (0 - 0) | | 0 (0 - 0) | 0 (0 - 0) |
| CXCL5 | 390 (62.3 - 732) | 198 (112 - 612) | 119 (101 - 785) | 129 (95.9 - 240) | | 52.6 (1.87 - 260) | 53.4 (5.84 - 167) |
| CXCL8 | 231 (71.7 - 1150) | 252 (89.3 - 337) | 266 (218 - 1830) | 302 (158 - 9270) | | 158 (71.1 - 356) | 187 (76.7 - 300) |
| CXCL9 | 50.3 (31.3 - 240) | 49 (24.5 - 172) | 39.1 (29.3 - 41.3) | 76.4 (25.3 - 105) | | 41.8 (16.8 - 72.1) | 29.4 (25.5 - 73.3) |
| CXCL10 | 72.6 (43 - 233) | 85.7 (22.5 - 236) | 95 (68.2 - 263) | 170 (68 - 1040) | | 86.2 (40.9 - 137) | 93.1 (41 - 125) |
| CXCL13 | 16.6 (10.9 - 217) | 14.5 (10.2 - 249) | 24.1 (4.74 - 162) | 25.8 (6.18 - 667) | | 15.8 (13 - 33.5) | 23.8 (13.6 - 50.8) |
| XCL-1 | 10.2 (6.47 - 123) | 11.7 (8.91 - 51.5) | 11.2 (10.9 - 11.5) | 12.5 (12.4 - 16.9) | | 13.9 (7.16 - 17.6) | 11.4 (7.16 - 16.6) |
| **Adipokines** |  |  |  |  | |  |  |
| Adiponectin^C^ | 0.991 (0.699 - 1.23) | 1.05 (0.807 - 1.17) | 0.857 (0.617 - 0.906) | 0.938 (0.915 - 1.06) | | 0.951 (0.872 - 1.09) | 1.05 (1.02 - 1.07) |
| Adipsin^A^ | 102 (26.5 - 226) | 120 (28.9 - 159) | 64.6 (63.8 - 70.9) | 103 (88.8 - 130) | | 61.8 (22.6 - 162) | 73.4 (34.5 - 102) |
| Cathepsin B^A^ | 15.3 (1.76 - 20.2) | 17.5 (2.16 - 23.1) | 20.4 (10.5 - 26) | 13.3 (8.74 - 16.7) | | 9.64 (0.945 - 18.5) | 8.91 (2.15 - 22.6) |
| Cathepsin L^A^ | 14.1 (7.74 - 17.7) | 9.77 (4.9 - 18.3) | 6.78 (4.62 - 7.95) | 5.54 (5.3 - 17) | | 10.4 (6.75 - 12.3) | 7.03 (5.53 - 8.26) |
| Cathepsin S^A^ | 309 (259 - 347) | 279 (225 - 362) | 227 (191 - 258) | 269 (231 - 316) | | 302 (288 - 335) | 251 (203 - 284) |
| Chemerin^B^ | 1.31 (0.974 - 1.58) | 1.43 (0.897 - 1.82) | 0.768 (0.467 - 0.96) | 1.3 (1.03 - 1.39) | | 1.39 (1.03 - 2.09) | 1.43 (1.23 - 2.09) |
| Leptin^B^ | 0.496 (0.345 - 1.66) | 0.336 (0.141 - 1.23) | 0.198 (0.142 - 0.258) | 0.189 (0.12 - 0.2) | | 0.519 (0.246 - 1.05) | 0.288 (0.162 - 1.67) |
| Omentin | 0.01 (0 - 0.01) | 0.01 (0 - 0.01) | 0.01 (0.01 - 0.01) | 0.01 (0 - 0.01) | | 0.01 (0 - 0.01) | 0 (0 - 0.01) |
| PAI-1^B^ | 4.02 (3.49 - 5.17) | 3.43 (2.97 - 4.53) | 3.71 (2.99 - 3.84) | 3.86 (2.73 - 5.17) | | 3.62 (3.42 - 5.41) | 3.52 (3.2 - 3.97) |
| RBP-4^C^ | 0.389 (0.326 - 0.571) | 0.325 (0.314 - 0.352) | 0.393 (0.322 - 0.479) | 0.317 (0.288 - 0.576) | | 0.312 (0.264 - 0.417) | 0.342 (0.312 - 0.396) |
| Resistin^B^ | 1.11 (0.855 - 1.3) | 1.21 (0.973 - 1.38) | 1.33 (1.03 - 1.33) | 1.26 (1.21 - 1.51) | | 1.23 (0.895 - 1.35) | 1.09 (1.08 - 1.2) |
| SAA-1^A^ | 103 (0 - 226) | 131 (29.4 - 235) | 148 (0 - 302) | 194 (19.5 - 472) | | 194 (134 - 235) | 162 (42.8 - 206) |
| TIMP-1^A^ | 381 (311 - 482) | 369 (300 - 419) | 323 (311 - 363) | 317 (309 - 431) | | 402 (328 - 417) | 359 (354 - 373) |
| Trombopoietin^B^ | 1.46 (1.23 - 3.65) | 2.09 (1.26 - 3.95) | 1.1 (0.59 - 1.11) | 1.98 (0.755 - 2.87) | | 1.16 (0.348 - 2.1) | 0.986 (0.327 - 1.68) |
| **Growth factors** |  |  |  |  | |  |  |
| BDNF | 257 (104 - 703) | 148 (105 - 209) | 308 (71 - 413) | 116 (62.6 - 171) | | 108 (59.6 - 143) | 99.6 (75.9 - 144) |
| EGF | 58.7 (28.9 - 199) | 56.7 (4.92 - 93.6) | 44.4 (16.4 - 71.2) | 25.3 (19.6 - 37.2) | | 69.7 (22.6 - 80.2) | 44.1 (14 - 74.6) |
| G-CSF^A^ | 58.7 (30.9 - 73) | 50.3 (28 - 83.9) | 66 (45.5 - 72.3) | 64 (43.5 - 67.1) | | 50.6 (40.3 - 140) | 48.5 (41.3 - 127) |
| GM-CSF | 3.42 (0 - 4.85) | 1.85 (0 - 4.01) | 0 (0 - 91.5) | 9.41 (0 - 372) | | 6.15 (0 - 14.5) | 1.21 (0 - 4.53) |
| HGF^A^ | 1.36 (0.507 - 3.53) | 1.38 (0.511 - 2.44) | 0.949 (0.767 - 1.56) | 1.08 (0.604 - 4.03) | | 1.13 (0.122 - 1.51) | 0.933 (0.224 - 1.08) |
| M-CSF | 37.1 (21.7 - 54.9) | 30.5 (24.2 - 40.5) | 35.8 (31.6 - 44.3) | 31.2 (29.8 - 31.5) | | 27.3 (24.3 - 32) | 28.1 (25.2 - 29.1) |
| NGF | 0 (0 - 0) | 0 (0 - 0) | 0 (0 - 0) | 0 (0 - 0) | | 0 (0 - 0) | 0 (0 - 0) |
| SCF | 15.3 (4.77 - 35.6) | 16.4 (4.77 - 18.5) | 8.53 (7.87 - 13) | 10.4 (9.06 - 10.5) | | 6.52 (4.77 - 7.37) | 5.46 (4.48 - 10.9) |
| sICAM^A^ | 68.6 (43.8 - 110) | 73 (51.4 - 87.3) | 77.5 (75.2 - 195) | 89.5 (74.4 - 349) | | 104 (85.6 - 124) | 83.1 (72.9 - 104) |
| sVCAM^B^ | 0.787 (0.399 - 1.14) | 0.517 (0.33 - 0.686) | 0.519 (0.327 - 0.804) | 0.655 (0.577 - 1.27) | | 0.614 (0.483 - 0.805) | 0.52 (0.436 - 0.61) |
| VEGF^A^ | 1.41 (1.13 - 2.03) | 1.26 (0.543 - 2.77) | 1.7 (0.841 - 3.56) | 1.96 (0.574 - 2.88) | | 1.11 (0.385 - 1.33) | 1.02 (0.817 - 1.36) |
| **Other** |  |  |  |  | |  |  |
| FAS | 595 (305 - 1820) | 685 (550 - 1490) | 586 (369 - 647) | 770 (673 - 814) | | 415 (54.3 - 811) | 408 (83.5 - 1100) |
| FAS-L | 6.82 (4.57 - 10.7) | 6.3 (5.84 - 8.66) | 6.83 (5.29 - 7.52) | 7.2 (5.55 - 8.53) | | 5.65 (4.81 - 7.94) | 7.12 (6.06 - 8.53) |
| Granzyme B | 39.8 (21.8 - 53.3) | 43.1 (23.4 - 56.8) | 50.6 (37.3 - 59.3) | 48.2 (36.9 - 110) | | 41.5 (25.9 - 44) | 33.3 (28.8 - 53.7) |
| IL-1RII^A^ | 6.35 (2.12 - 7.33) | 4.98 (2.29 - 9.17) | 5.41 (5.4 - 6.16) | 5.49 (4.58 - 7.46) | | 1.54 (1.22 - 3.49) | 2.11 (1.14 - 3.37) |
| IL-18BPA | 157 (66 - 235) | 158 (82.1 - 279) | 209 (146 - 1000) | 223 (160 - 5310) | | 114 (66.2 - 149) | 102 (80.8 - 271) |
| KIM_1 | 19.9 (5.92 - 37.1) | 45.6 (20.1 - 161) | 20.4 (7 - 39.7) | 23.3 (8.82 - 30.4) | | 12.4 (6.57 - 54.1) | 12.9 (5.04 - 17.1) |
| MMP-8 | 87.4 (81.1 - 473) | 86.5 (73.3 - 98.9) | 83.5 (69.6 - 84.4) | 80.8 (64.9 - 85.3) | | 81.1 (74.6 - 98) | 91.5 (74.5 - 106) |
| OPG | 648 (411 - 837) | 595 (525 - 784) | 441 (299 - 657) | 512 (328 - 524) | | 395 (103 - 688) | 392 (227 - 516) |
| OPN^A^ | 2.23 (0.861 - 3.38) | 2.61 (1.15 - 4.26) | 2.84 (1.45 - 5.22) | 3.37 (3.35 - 3.41) | | 1.62 (0 - 3.32) | 1.56 (0 - 5.46) |
| S100A12^A^ | 178 (97 - 504) | 103 (35.7 - 303) | 563 (151 - 566) | 273 (226 - 449) | | 146 (37.6 - 187) | 37.3 (25.4 - 136) |
| sCD14^A^ | 90.9 (68.5 - 10100) | 96.8 (64.2 - 156) | 78.7 (47.1 - 84.1) | 82 (60.7 - 85.1) | | 94.4 (78.4 - 10100) | 67 (65.1 - 68.2) |
| sCD25 | 633 (294 - 785) | 662 (347 - 760) | 767 (619 - 813) | 700 (655 - 1480) | | 632 (414 - 715) | 519 (485 - 833) |
| sCD163^A^ | 14.9 (2.6 - 18.1) | 12.3 (2.06 - 30.2) | 14.8 (13.3 - 15.3) | 17 (11.2 - 44.5) | | 8.09 (4.48 - 16.3) | 7.82 (6.51 - 14.4) |
| sIL-6R^A^ | 1.98 (0.793 - 2.26) | 1.75 (0.947 - 2.68) | 1.99 (1.51 - 2.18) | 1.68 (1.36 - 1.95) | | 1.62 (0.354 - 1.84) | 1.39 (0.581 - 2.47) |
| sPD-1 | 250 (112 - 1700) | 172 (51.8 - 910) | 164 (107 - 263) | 128 (84.2 - 158) | | 188 (3.3 - 376) | 64.6 (10.1 - 247) |
| sSCF-R^A^ | 40.5 (11 - 40.5#) | 38.9 (12.1 - 40.5#) | 34.9 (34.1 - 40.5#) | 40.5 (25.8 - 46.2) | | 18.4 (2.4 - 28) | 19.8 (5.76 - 46.8) |
| TNF-RI^A^ | 3.06 (1.08 - 4.46) | 3.86 (1.58 - 4.1) | 1.71 (1.55 - 3.37) | 2.24 (0.742 - 4.1) | | 2.59 (0.833 - 5.66) | 2.93 (1.66 - 5.25) |
| TNF-RII^A^ | 1.09 (0.654 - 2.08) | 1.32 (1.03 - 2.58) | 1.2 (1.17 - 2.07) | 1.69 (1.47 - 1.7) | | 1.12 (0.694 - 1.81) | 1.04 (0.725 - 3.01) |
| TREM-1 | 11.8 (3.89 - 32.6) | 7 (5.15 - 14) | 9.43 (6.3 - 12.9) | 8.62 (6.19 - 11.2) | | 5.96 (5.15 - 9.08) | 8.36 (5.5 - 10.6) |
